# Outcomes of ventricular tachycardia ablation in patients with structural heart disease: The impact of electrical storm

**DOI:** 10.1371/journal.pone.0171830

**Published:** 2017-02-10

**Authors:** Bashar Aldhoon, Dan Wichterle, Petr Peichl, Robert Čihák, Josef Kautzner

**Affiliations:** Department of Cardiology, Institute for Clinical and Experimental Medicine – IKEM, Prague, Czech Republic; University of Minnesota, UNITED STATES

## Abstract

**Aims:**

To investigate predictors of long-term outcomes after catheter ablation (CA) for ventricular tachycardia (VT) and the impact of electrical storm (ES) prior to index ablation procedures.

**Methods:**

We studied consecutive patients with structural heart disease and VT (n = 328; age: 63±12 years; 88% males; 72% ischaemic cardiomyopathy; LVEF: 32±12%) who had undergone CA. According to presenting arrhythmia at baseline, they were divided into ES (n = 93, 28%) and non-ES groups. Clinical predictors of all-cause mortality were investigated and a clinically useful risk score (SCORE) was constructed.

**Results:**

During a median follow-up of 927 days (IQR: 564–1626), 67% vs. 60% of patients (p = 0.05) experienced VT recurrence in the ES vs. the non-ES group, respectively; and 41% vs. 32% patients died (p = 0.02), respectively. Five factors were independently associated with mortality: age >70 years (hazard ratio (HR): 1.6, 95% confidence interval (CI): 1.1–2.4, p = 0.01), NYHA class ≥3 (HR: 1.9, 95% CI: 1.2–2.9, p = 0.005), a serum creatinine level >1.3 mg/dL (HR: 1.6, 95% CI: 1.1–2.3, p = 0.02), LVEF ≤25% (HR: 2.4, 95% CI: 1.6–3.5, p = 0.00004), and amiodarone therapy (HR: 1.5, 95% CI: 1.0–2.2, p = 0.03). A risk SCORE ranging from 0–4 (1 point for either high-risk age, NYHA, creatinine, or LVEF) correlated with mortality. ES during index ablation independently predicted mortality only in patients with a SCORE ≤1.

**Conclusions:**

Advanced LV dysfunction, older age, higher NYHA class, renal dysfunction, and amiodarone therapy, but not ES, were predictors of poor outcomes after CA for VT in the total population. However, ES did predict mortality in a low-risk sub-group of patients.

## Introduction

Previous studies suggest that electrical storm (ES) is a life-threatening condition with specific management issues and poor prognosis compared to sporadic ventricular tachycardia (VT) episodes [1]. ES mainly affects patients with structural heart disease (SHD), of both ischaemic and non-ischaemic aetiology. Over the last 10–15 years, catheter ablation (CA) has emerged as an effective treatment modality in patients with ES [2]. In a proportion of cases it may even be considered a life-saving procedure [3,4]. Recent meta-analysis shows that CA of ES has high-acute success rates, with a low rate of recurrent storms [5]. However, there is a lack of information on the long-term outcomes and predictors of survival after CA for ES [6,7]. We aimed to investigate the differences between patients ablated for ES and non-ES ventricular arrhythmia, and to assess long-term outcomes in terms of arrhythmia recurrence and all-cause mortality in patients with SHD.

## Methods

### Patient population and study design

We studied consecutive patients with SHD-related VT who underwent CA in our centre between August 2006 and August 2013. ES was defined as the occurrence of 3 or more distinct episodes of VT within 24 hours, requiring the intervention of either an implantable or external defibrillator [8]. Patients with incessant VT were also considered as having ES. Two sub-groups were defined according to the presence or absence of ES during the first ablation procedure (the ES group vs. the non-ES group). All potential reversible causes of VT were excluded and various measures, including beta-blocker, amiodarone, and/or deep sedation and mechanical ventilation were used when indicated to stabilise clinical status before CA. We used pre-procedural transthoracic or transesophageal echocardiography to evaluate cardiac anatomy and function, and to rule out intracavitary thrombosis in all cases.

We analysed clinical characteristics, long-term outcomes, and outcome predictors. For this purpose, 2 clinical endpoints were defined: first VT recurrence, and all-cause mortality.

### Catheter ablation procedure

CA was performed as previously described [9]. In brief, the procedure was performed under conscious sedation, except for patients who required mechanical ventilation as part of ES management. The left ventricle was entered either retrogradely or transseptally, depending on the presumed substrate location and other factors, such as INR level, peripheral arterial disease, aortic valve stenosis, or mechanical prosthesis. Intravenous heparin was administered to maintain the activated clotting time ≈300–350 seconds.

Except for incessant VT, programmed ventricular stimulation was initially performed with two basic stimulation trains (cycle length of 600 and 400 ms) and up to three extrastimuli. The protocol was repeated when the operator judged that the extent of the ablation was sufficient to prevent clinical VT and that the arrhythmogenic substrate was adequately ablated. In all patients in whom VT with a cycle length ≥250 ms were still inducible, further ablation lesions were deployed. Final testing for VT inducibility was left to the discretion of the operator based on clinical manifestation of arrhythmia, the extent of the substrate and ablation, the procedure duration, patient characteristics, and haemodynamic status.

All subjects underwent three-dimensional electroanatomic mapping (CARTO, Biosense Webster, Diamond Bar, CA) to define scars or areas of low voltage using a 3.5-mm open-irrigated-tip catheter (Navistar Thermocool, Biosense Webster). Late potentials were tagged and pacing was used to assess slow conduction zones. RF energy applications primarily targeted the more central, slowly conducting channels. Successful deployment of the lesions was confirmed by non-capture at given sites. Additional lesions were applied in areas of late potentials for substantial arrhythmogenic substrate modification. In haemodynamically stable patients we attempted to eliminate all inducible VT. In vulnerable patients we targeted clinical VT. In unstable patients with incessant VT our endpoint was the restoration of stable sinus rhythm.

### Clinical follow-up

The vast majority of patients (93%) were routinely evaluated in our outpatient clinic at 3- to 6-month intervals. All complications were documented using a multi-level departmental tracking process. Major complications were defined as those that required specific intervention or a prolonged hospital stay due to complications (a median of 5 days after ablation). For patients not followed at our hospital, referring hospitals were contacted and records were reviewed to confirm VT recurrence as well as heart transplant and ventricular assist device-free survival. Data on mortality were obtained/verified for all study subjects from the national registry of citizens.

### Statistical analysis

Continuous variables were expressed as means with standard deviations and compared using the 2-tailed t-test for independent samples. Clearly non-normally distributed variables were expressed as medians and interquartile ranges (IQR) and compared using the Mann-Whitney U test. Categorical variables were expressed as percentages and compared using the Chi-square test. Follow-up data were censored at the time of death, heart transplant, implantation of ventricular assist device, or the last contact; whichever came first. Event-free survival was analysed using Kaplan-Meier curves and a log-rank test. Factors associated with the risk of clinical events were identified using Cox regression models of proportional hazards. Variables subjected to univariate screening included: ES at index ablation, age >70 years, female gender, ischaemic CMP, NYHA class ≥3, LVEF ≤25%, left ventricular end-diastolic diameter >60 mm, serum creatinine >1.3 mg/dL, and specific antiarrhythmic or amiodarone treatment. Variables showing significant or marginal associations (p<0.10) with clinical events under univariate analysis were assessed using multivariate models. A clinical scoring system was employed for the prediction of all-cause mortality. P-values ≤0.05 were considered significant. All analyses were performed using STATISTICA version 12 software (Statsoft, Inc., Tulsa, USA).

## Results

### Patient characteristics

Of a total of 328 patients with SHD undergoing VT ablation (466 procedures), 93 patients (28%) belonged to the ES group. Patients in the entire population received a median of 3 shocks (1–7) during the last seven days prior to CA.

Baseline characteristics of patients from the ES and non-ES groups are shown in Table 1. In comparison with non-ES patients, the ES group presented with significantly higher incidence of heart failure, higher NYHA class, lower LVEF, higher incidence of renal dysfunction (defined as a level of serum creatinine >1.3 mg/dL), and a higher proportion of previously implanted ICDs or resynchronisation therapy devices.

**Table 1:** Baseline characteristics of the study population.

	ES patients (n = 93)	Non-ES patients (n = 235)	
	Mean±SD or median (IQR) or percentage	Mean±SD or median (IQR) or percentage	P
Age (yrs)	64.4±10.6	63.0±12.7	0.37
Age >70 yrs	24.7%	29.4.1%	0.40
Females	9.7%	12.3%	0.50
Hypertension	64.5%	58.7%	0.34
Heart failure	95.7%	82.1%	0.001
NYHA class	2.5±0.9	2.2±0.9	0.005
NYHA class ≥3	56.5%	41.2%	0.01
Diabetes	29.0%	27.7%	0.80
Stroke/transient ischaemic attack	12.9%	8.1%	0.18
Coronary artery disease	77.4%	71.1%	0.24
Peripheral vascular disease	14.0%	12.8%	0.77
Creatinine (mg/dL)	1.2 (1.1–1.5)	1.1 (1.0–1.4)	0.003
Creatinine >1.3 mg/dL	45.2%	31.5%	0.02
LV ejection fraction (%)	28.0±9.0	34.3±12.3	0.00001
LV ejection fraction ≤25%	57.0%	34.5%	0.0002
LV end-diastolic diameter (mm)	65.7±8.3	64.8±9.6	0.41
LV end-diastolic diameter >60 mm	69.6%	66.2%	0.57
Ischemic CMP	76.3%	70.6%	0.30
Dilated CMP	19.3%	15.7%	0.43
Arrhythmogenic right ventricular CMP	1.1%	7.2%	0.03
Hypertrophic obstructive CMP	0.0%	0.9%	0.37
Arrhythmia-induced CMP	0.0%	2.1%	0.16
Inflammatory CMP	3.2%	0.9%	0.11
Spongious CMP	0.0%	1.3%	0.28
Congenital CMP	0.0%	0.9%	0.37
Valvular CMP	1.1%	1.7%	0.68
Other CMP	3.2%	2.1%	0.56
Implantable cardioverter-defibrillator	91.4%	82.1%	0.04
Cardiac resynchronisation therapy	45.2%	31.1%	0.02
Class I or III antiarrhythmic drugs	55.9%	49.6%	0.30
Amiodarone	47.3%	43.2%	0.50

Abbreviations: CMP, cardiomyopathy; ES, electrical Storm; LV, left ventricular; NYHA, New York Heart Association.

### Catheter ablation procedure

Table 2 shows the procedural characteristics of both patient groups in “per procedure” fashion. The procedures for ES were more likely to be non-elective and were preceded by a higher number of shocks as well as by more frequent cardiopulmonary resuscitation prior to the procedure.

**Table 2:** Procedural characteristics of the study population.

	ES ablation(n = 139)	Non-ES ablation(n = 327)	
	Mean±SD or median (IQR) or percentage	Mean±SD or median (IQR) or percentage	P
Prior cardiopulmonary resuscitation	8.6%	2.8%	0.005
Shocks ≤7 days prior to ablation	3 (1–7)	0 (0–1)	<0.00001
Transseptal access	8.6%	9.5%	0.77
Epicardial access	3.6%	5.5%	0.39
Endocardial right ventricular ablation	13.7%	21.7%	0.04
Endocardial left ventricular ablation	89.2%	81.3%	0.04
Epicardial ablation	2.2%	3.7%	0.40
Intracardiac echocardiography	25.2%	34.9%	0.04
Major complication	10.1%	7.6%	0.39
Major vascular complication	4.3%	4.9%	0.79
Major non-vascular complication	5.8%	2.8%	0.11
Radiofrequency time (sec)	1535±899	1389±1017	0.15
Fluoroscopic time (min)	14.4±7.5	16.3±8.8	0.03
Procedure time (min)	201±65	210±61	0.16
Elective procedure	2.2%	30.3%	<0.00001

Abbreviations: ES, electrical storm.

In 88 of the procedures (19%), the final programmed ventricular stimulation was not performed mainly due to initial VT non-inducibility. Other reasons were due to the length of the procedure or because of safety concerns in haemodynamically unstable patients. Programmed ventricular stimulation was applicable in 378 of the procedures (81%), of which non-inducibility of clinical VT was achieved in 282 procedures (75%) [78 (74%) and 204 (75%) in the ES and non-ES group, respectively]. The non-inducibility of any VT was achieved in 223 procedures (59%) [56 (53%) and 167 (61%) in the ES group and non-ES group, respectively]. In 155 procedures (41%) with positive last interim inducibility testing, additional lesions were deployed in 140 procedures (37%) [46 (44%) and 94 (34%) in the ES and non-ES group, respectively)], but complete final testing was not performed. The reasons were due to either the length of the procedure or the haemodynamic status of the patient.

### Complications

The rates of major procedure-related complications, either vascular or non-vascular, were not significantly different between the study groups, although there was a trend towards higher occurrence of complications in the ES group (Table 2). Importantly, no peri-procedural deaths occurred. Non-vascular complications included 3 cases of haemopericardium, 3 strokes, 2 transitory ischaemic attacks, 1 thromboembolic event to the left lower limb, 2 cardiac arrests, 1 case of post-ablation pericarditis, 4 cases of conduction system damage and 1 case of RV pacing lead dysfunction due to focal ablation adjacent to the lead. Vascular complications included 14 pseudoaneurysms, 5 arteriovenous fistulas, 1 femoral artery injury, and 2 cases of major groin bleeding.

### Clinical follow-up

#### Arrhythmia recurrences

**Fig 1:**
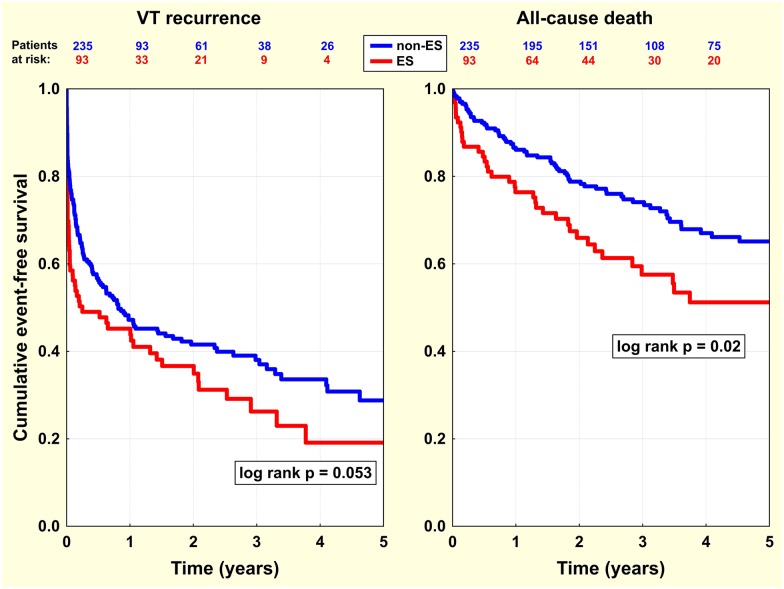
Event-free survival analysis in ES versus non-ES patients. Kaplan-Meier curves show event-free survival in the 2 main study groups—in patients who were ablated for ES (red line) versus non-ES ventricular arrhythmia (blue line). Separate graphs and comparisons were assembled for individual endpoints—VT recurrence after index ablation (left panel), and all-cause death after index ablation (right panel). Note that ES patients had worse outcomes for both endpoints.

During a median follow-up of 927 days (interquartile range 564–1626), the VT recurrence rate reached 62/93 patients (66.7%) in the ES group vs. 141/235 patients (60.0%) in the non-ES group (log rank p = 0.053) (Fig 1), including 38/93 patients (40.9%) vs. 58/235 patients (24.7%) (log rank p = 0.003) who had recurrences within the first 30 days. 12-month cumulative event-free survival was 41/93 patients (44.1%) and 112/235 patients (47.7%) (p = 0.56) in the ES and non-ES group, respectively. The rate of repeated ablation was significantly higher in the ES group, with 33/93 patients (35.5%) vs. 65/235 patients (27.7%) (log rank p = 0.04). Patients in the ES group underwent a mean of 1.45±0.71 ablations compared to 1.41±0.81 in the non-ES group (p = 0.66). The median (IQR) time for the first repeated ablation for all patients (n = 98) was 50 days (7–212) with a significantly shorter period for 33 patients in the ES group compared to 65 patients in the non-ES group [8 (3–94) vs. 72 days (21–269), p = 0.002, respectively]. The first repeated ablation was indicated for ES in 18/93 patients (19%) and 18/33 procedures (55%) in the ES group, and 12/235 patients (5%) and 12/65 procedures (18%) in the non-ES group (p = 0.0001 and p = 0.0002). Patients with post-procedural VT non-inducibility had non-significantly less VT recurrences during follow-up compared to the rest of the population (log rank p = 0.11).

#### All-cause mortality

Patients from the ES group had significantly higher all-cause mortality than those in the non-ES group [38/93 (40.9%) vs. 75/235 patients (31.9%), log rank p = 0.02] (Fig 1). 12-month cumulative event-free survival for all-cause mortality was 71/93 patients (76.3%) vs. 202/235 patients (86.0%) (p = 0.03) in the ES and non-ES group, respectively. Seven patients (8%) in the ES group underwent heart transplantation and 4 (4%) received ventricular assist devices compared to 10 transplants (4%) and 12 ventricular assist devices (5%) in the non-ES group. Transplants and ventricular assist device implants were performed predominantly for end-stage heart failure and, only in 4 patients in the whole patient population, for uncontrollable ventricular arrhythmias. Four patients had VADs implanted prior to ablation, while 3 patients received VADs within one week and the remaining patients within eight months after the last ablation. Heart transplantation was performed 3–32 months after the last ablation depending on the presence of a suitable donor.

### Outcome predictors

Table 3 shows the univariate association of baseline factors with: 1) VT recurrence after index CA and 2) total mortality. ES during index ablation was weakly associated with VT recurrence and all-cause mortality, while high NYHA class and low LVEF were the strongest predictors of these clinical events. Under multivariate analysis (Table 4), the initial occurrence of ES appeared to be a non-significant predictor of adverse events. Advanced age, high NYHA class, low LVEF, renal function impairment, and amiodarone treatment at baseline remained independent predictors of total mortality.

**Table 3:** Univariate survival analysis.

Endpoint:	VT/VF recurrence after index ablation			All-cause death		
Factor	HR	95% CI	P	HR	95% CI	P
ES group	1.4	1.0–1.8	0.045	1.6	1.1–2.4	0.02
Age >70 yrs	1.0	0.7–1.4	0.96	1.6	1.1–2.4	0.01
Female gender	1.3	0.8–1.9	0.30	0.7	0.4–1.4	0.36
Ischaemic CMP	1.0	0.7–1.3	0.79	2.0	1.2–3.2	0.007
NYHA class ≥3	1.7	1.2–2.2	0.0004	3.3	2.2–4.9	<0.00001
LVEF ≤25%	1.6	1.2–2.2	0.0006	3.0	2.1–4.4	<0.00001
LVEDd >60 mm	1.4	1.0–1.9	0.03	1.9	1.2–2.9	0.005
Creatinine > 1.3 mg/dL	1.0	0.8–1.4	0.79	2.4	1.6–3.4	<0.00001
Class I or III AADs	1.0	0.8–1.4	0.75	1.8	1.2–2.7	0.002
Amiodarone	1.0	0.8–1.4	0.82	2.0	1.4–2.9	0.0003

Cox proportional hazard ratios (HR) with 95% confidence intervals (CI) for the 2 study endpoints are shown for dichotomised baseline factors.

Abbreviations: AADs, anti-arrhythmic drugs; CMP, cardiomyopathy; ES, electrical storm; LVEF, left ventricular ejection fraction; LVEDd, left ventricular end-diastolic diameter; NYHA, New York Heart Association. Drug treatment factors relate to the time of individual events (arrhythmia recurrence or death).

**Table 4:** Multivariate survival analysis.

Endpoint:	VT/VF recurrence after index ablation			All-cause death		
Factor	HR	95% CI	P	HR	95% CI	P
Age >70 yrs				1.6	1.1–2.4	0.01
NYHA class ≥3	1.5	1.1–2.0	0.01	1.9	1.2–2.9	0.005
LVEF ≤25%	1.4	1.1–1.9	0.02	2.4	1.6–3.5	0.00004
Creatinine >1.3 mg/dL				1.6	1.1–2.3	0.02
Amiodarone				1.5	1.0–2.2	0.03

For the legend, see Table 3.

**Fig 2:**
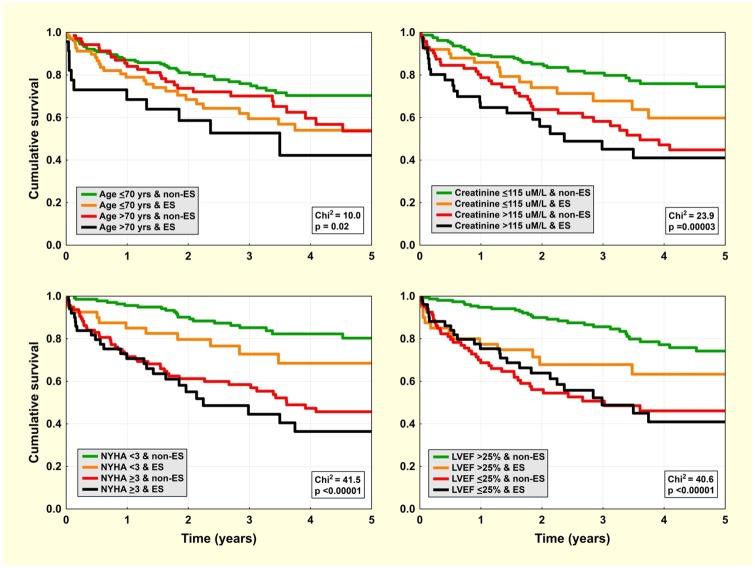
All-cause mortality—impact of individual clinical factors. Kaplan-Meier survival curves for the population dichotomised by ES/non-ES index ablation and by individual clinical factors: age ≤70 yrs (left upper panel), NYHA class <3 (left lower panel), serum creatinine ≤1.3 mg/dL (right upper panel), and LVEF ≤25% (right lower panel).

Mortality curves for selected dichotomised predictors in combination with ES/non-ES categories (in 2x2 factorial design) are displayed in Fig 2. The clinical risk score (ranging from 0–4) was computed as the sum of points for the following factors: age >70 yrs, LVEF ≤25%, NYHA class ≥3, and creatinine level >1.3mg/dL (for simplification purposes, 1 point for every factor). Amiodarone was excluded from the SCORE calculation because baseline amiodarone treatment is not an optimum factor in terms of statistics, i.e. while other clinical factors are relatively stable during long-term follow-up, amiodarone may be later discontinued because of adverse events or initiated because of arrhythmia recurrence. This may interfere with valid analysis. Additionally, amiodarone usage is dependent on treatment strategies in different regions/countries, which means that the inclusion of amiodarone as a factor would decrease the generalisability of the SCORE.

**Fig 3:**
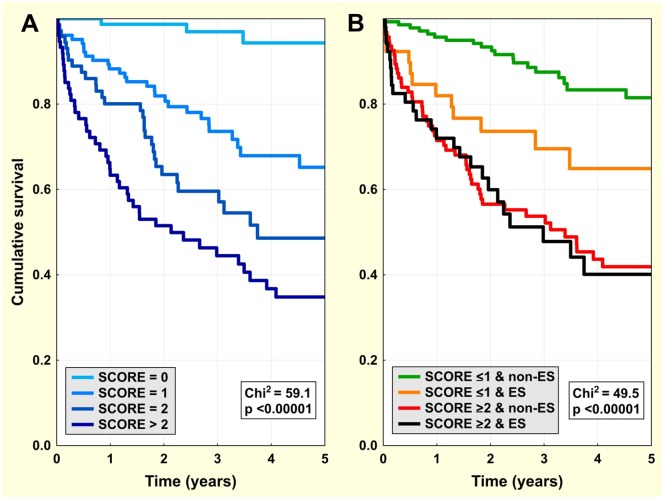
All-cause mortality—impact of clinical risk score. Kaplan-Meier survival curves for the population categorised by clinical risk score (Panel A) and in sub-groups defined by the combination of the main study groups (ES/non-ES) and dichotomised clinical risk scores (≤1 or ≥2) (Panel B).

There were 76, 103, 73, 64, and 12 patients with a SCORE of 0, 1, 2, 3, and 4, respectively. The clinical risk score was associated with a progressive increase of all-cause mortality (Fig 3A). When the population was dichotomised according to the SCORE (dichotomised at ≤1 because this best approximates the median SCORE, with all other dichotomies for individual factors also set at median where possible) and combined with ES/non-ES allocation (Fig 3B), ES during index ablation remained a significant risk factor of total mortality, but only in the low-risk sub-group with a SCORE ≤1 (Fig 3B and Table 5). In this sub-group (179 patients: 39 from the ES group and 140 from the non-ES group), ES at index ablation was associated with total mortality (HR: 2.5; 95% CI: 1.2–5.2; p = 0.02) independent of other clinical confounders.

**Table 5:** All-cause mortality in study groups stratified by clinical risk score.

	SCORE ≤1	SCORE ≥2	Comparison of mortality for patients with a high- vs. low-risk SCORE(HR, 95% CI, P)	
			for sub-groups	overall
Non-ES group	15.7%	55.8%	4.9 (3.0–8.0)P<0.00001	3.9 (2.6–5.8)p<0.00001
ES group	30.8%	48.1%	1.9 (1.0–3.8)p = 0.06	
Comparison of mortality for ES vs. non-ES patients(HR, 95% CI, P)	2.4 (1.2–4.9) p = 0.01	1.0 (0.64–1.6)p = 0.91		

Percentages indicate all-cause mortality during total follow-up stratified by clinical risk score (SCORE). Cox proportional hazard ratios (HR) with 95% confidence intervals (CI) for all-cause mortality are shown.

Abbreviations: ES, electrical storm.

30-day recurrence of VT appeared to be associated with higher long-term mortality (HR: 1.6; 95% CI: 1.1–2.4; p = 0.01) in the total population and the risk was comparable in both the ES and non-ES groups.

## Discussion

The present study provides an analysis of long-term outcomes of CA for VTs in patients with SHD with or without ES. The most important findings of this study are as follows:

1) Patients in the ES group had a higher prevalence of heart failure, lower LVEF, a worse NYHA class, and a higher level of serum creatinine; 2) the rate of re-do ablations was higher in the ES group with significantly shorter time for re-ablation; 3) patients in the ES group had significantly higher all-cause mortality, although ES was identified as an adverse prognostic factor but only in the sub-group of subjects with a low risk profile; 4) older age, more severe LV dysfunction, higher NYHA class, impaired renal function, and amiodarone treatment prior to ablation were identified as independent predictors of poor long-term outcomes after VT ablation.

### Triggers of electrical storm

Previous observations have suggested several factors that may facilitate ES. Severely compromised LV function, impairment of renal function, older age, ischaemia, hypokalaemia or hyperkalaemia, and infection have been identified as potential factors that trigger ES [10–12]. In agreement with these reports, we found that ES patients had a higher prevalence of heart failure, lower LVEF, a worse NYHA class, and a higher level of serum creatinine. We suppose that an occurrence of these factors may contribute to the development of ES. ES could thus be viewed as an epiphenomenon that itself indicates overall poor health status. However, in a subgroup of patients with no or one of the above risk factors, ES could be viewed as an indicator of electrical instability and predictor of mortality (Fig 3B and Table 5).

### Recurrences of ventricular arrhythmias after catheter ablation

In agreement with previously published studies [7,13], the overall recurrence rate of VT after index CA was considerably high during long-term follow-up. Long-term success in these types of patients seems to be moderate even when procedures are performed by experienced electrophysiologists. The relatively high VT recurrence rate reflects both the presence of a large myocardial substrate and difficulties in its modification.

Interestingly, we found an accumulation of VT recurrences in the ES group during the first 30 days after index ablation. It may be possible that the worsening of the clinical status of the patient contributes to the occurrence of VT in clusters. On the other hand, successful management of VT clusters may suppress VT over the long term [4]. This in turn may have a favourable impact on long-term survival. In line with this hypothesis, we found ES patients who were free of VT during the first 30 days after CA had just as favourable long-term survival as non-ES patients.

The above data on the detrimental consequences of ES may be viewed as an argument in favour of early intervention in cases of VT recurrence, a strategy studied both in the SMASH-VT [14] and the VTACH [13] trials. One might further propose purely prophylactic CA to be performed at the time of ICD implantation [15]. However, the effect of such an early intervention on long-term prognosis remains unknown.

### Predictors of ventricular arrhythmia recurrence

Previous studies have identified a number of risk factors that predict VT recurrence. These include failed CA, the presence of non-tolerated VT, history of heart failure, an increasing number of inducible VTs, history of atrial fibrillation, and post-ablation amiodarone therapy [16–18]. In the present study, we identified LVEF ≤25% and NYHA class ≥3 as independent predictors of VT recurrence after CA, which suggests that advanced heart failure is a trigger of VT. This is in accordance with Carbucicchio et al. [4]. In their study of a population of ES patients, advanced heart failure patients with cardiogenic shock were shown to be more prone to VT recurrence compared to those without cardiogenic shock. Our results suggest that prevention of further heart failure deterioration at the time of the first VT episode (potentially by optimising heart failure treatment or using resynchronisation therapy) might be of clinical value for preventing VT recurrence.

### Predictors of long-term survival after catheter ablation

We did not find ES to be an independent predictor of all-cause mortality. This result is in accordance with previous studies [1,10]. Conversely, Della Bella et al. [16] found that, 26 months after CA, cardiac mortality was independently associated with ES at the time of index ablation. The explanation for this discrepancy is probably due to lower mean LVEF (32.1±12 vs. 38.5±13%, respectively) and a higher proportion of both NYHA ≥III class (45.4 vs. 28.6%, respectively) and/or renal disease (35.3 vs. 22.2%, respectively) in our study. It implies that our patient cohort had more advanced heart disease and explains why ES was not a significant factor in the prediction of mortality. This explanation is also supported by our findings that ES is a predictor of all-cause mortality, but only in the sub-group of low-risk patients (Fig 3B). Similar to previous studies [16–18], we confirmed that low LVEF, a high NYHA class, advanced age, renal function impairment, and amiodarone therapy are predictors of all-cause mortality. These findings suggest that ES in high-risk patients is more likely a marker of health status deterioration. Furthermore, compared to low risk patients, high-risk patients may benefit less from CA over the long term in instances of both VT recurrence and mortality.

The role of post-procedural inducibility testing in long-term outcome predicting has been recently discussed [16,17,19,20]. These reports suggest that performing post-ablation non-inducibility testing may be of significant clinical value. We were not able to demonstrate an association between the results of post-procedural inducibility testing and clinical endpoints, even though rigorous non-inducibility testing was applicable in 81% of the procedures. Therefore, this diminished the ability of our study to detect the prognostic role of inducibility testing.

The assessment of risk factors (age, creatinine level, NYHA class, and LVEF) demonstrates the additive value of each risk factor in predicting all-cause mortality in patients with VT and SHD. Nevertheless, we were not able to derive the direct clinical value of the SCORE from our retrospective study. Scoring simply highlights that the prognosis of patients with ventricular arrhythmias (even with catheter ablation) is heavily influenced by their background vital status. This might be helpful when selecting patients for future randomised studies on ablation treatment of ventricular arrhythmias.

### Limitations

The present study has several limitations. First, it is a single-centre, retrospective, observational study that analyses long-term outcomes in patients with VT who have undergone CA, and does not compare them with patients treated conservatively. Second, acute procedural outcomes were not assessed rigorously in all patients, which means that the study provides inconclusive evidence on the predictive value of non-inducibility testing at the end of the ablation procedure. Third, in patients who died during follow-up we did not investigate the immediate cause of death as the majority of them died outside our hospital. Fourth, data on clinical VT characteristics (VT morphology) were not available for all subjects; therefore, we could not compare arrhythmias present at follow-up with the original clinical arrhythmias. Finally, the burden of arrhythmia therapies after ablation was not evaluated and, therefore, we could not examine the potential injurious effects of repetitive shocks.

## Conclusions

This study shows that patients with ES comprise a generally higher risk population and have more recurrences of ventricular arrhythmias and higher all-cause mortality after CA than patients ablated for sporadic episodes of VT. ES does not independently confer increased mortality in patients who have undergone CA due to VT. However, we did identify ES to be an adverse prognostic factor, but only in a sub-group of subjects with a low-risk profile. We identified advanced age, increased LV dysfunction, a higher NYHA class, impaired renal function, and amiodarone treatment prior to ablation as independent predictors of poor long-term outcomes after VT ablation.

## Supporting information

S1 Table Dataset. (XLSX)
